# Supplementary material for: The hybrid history of zebrafish
Source: G3 (Bethesda). 2024 Dec 19;15(2):jkae299. doi: 10.1093/g3journal/jkae299 (PMC11797037; doi:10.1093/g3journal/jkae299)
Supplement: jkae299_Supplementary_Data [file jkae299_supplementary_data.pdf]

Supplemental Figure S1: Pairwise split frequencies across each chromosome.
Proportions of the six pairwise splits in the *D. rerio* species group in sliding windows of 500 pairwise splits across each chromosome. The gray bar shows the 95% confidence interval under the null expectation of equal split proportions.

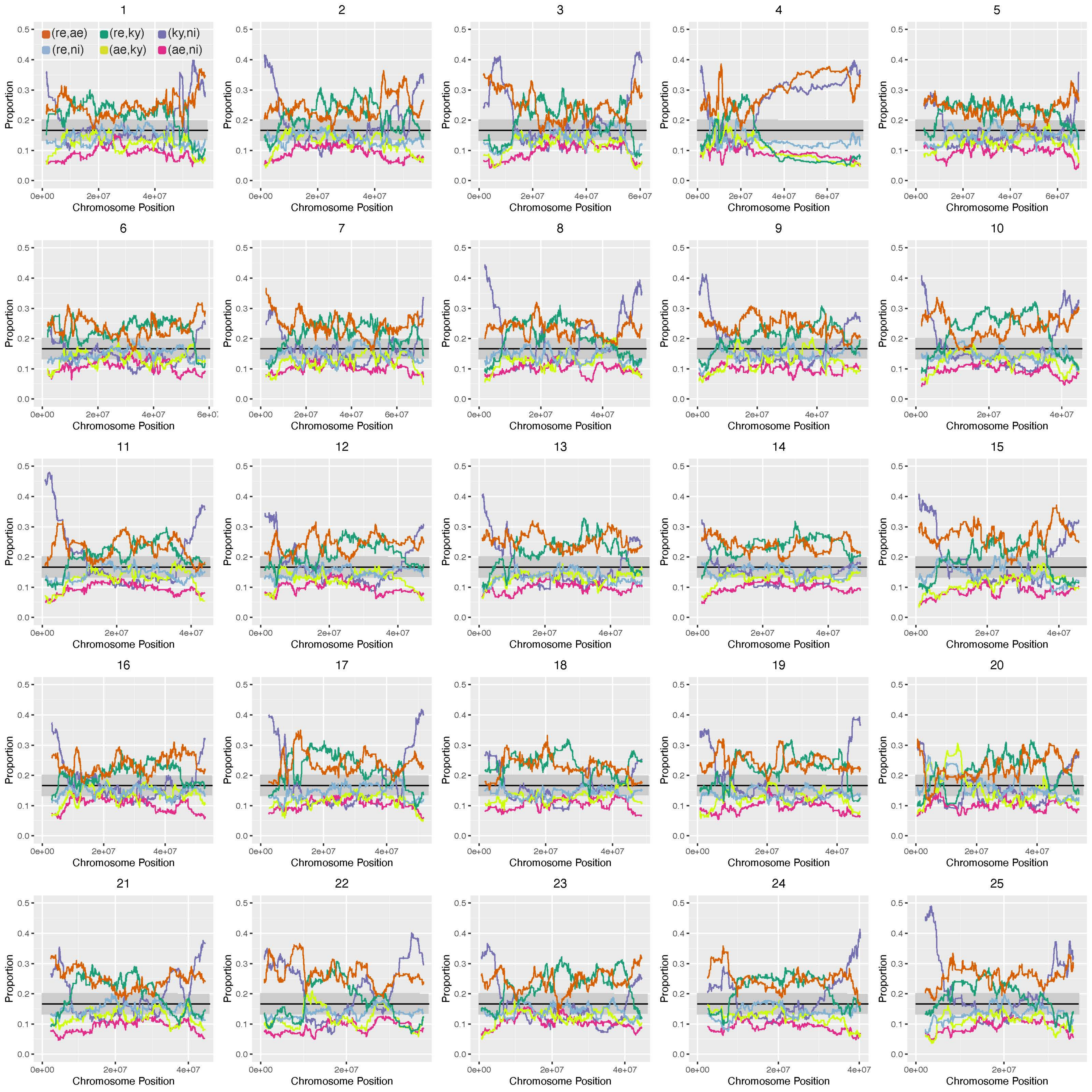
1
2
3
4
5
6
7
8
9
10
11
12
13
14
15
16
17
18
19
20
21
22
23
24
25
(re,ae)
(re,ky)
(ky,ni)
(re,ni)
(ae,ky)
(ae,ni)
Proportion
Chromosome Position
0.0
0.1
0.2
0.3
0.4
0.5
0e+00
2e+07
4e+07
6e+07

Supplemental Figure S2: Genome-wide distribution of genomic features and data used for phylogenetic inference (A) Histogram of SNPs in the *D. rerio* species group used in this study. Bins on the left are near the centers of chromosomes. Bins on the right are near the ends of chromosomes. (B) Histogram of annotated protein-coding genes in the zebrafish genome (GRCz10). (C) Histogram of RAD-seq loci used in (McCluskey and Postlethwait 2015). (D) Location of *SbfI* restriction sites in the zebrafish genome.

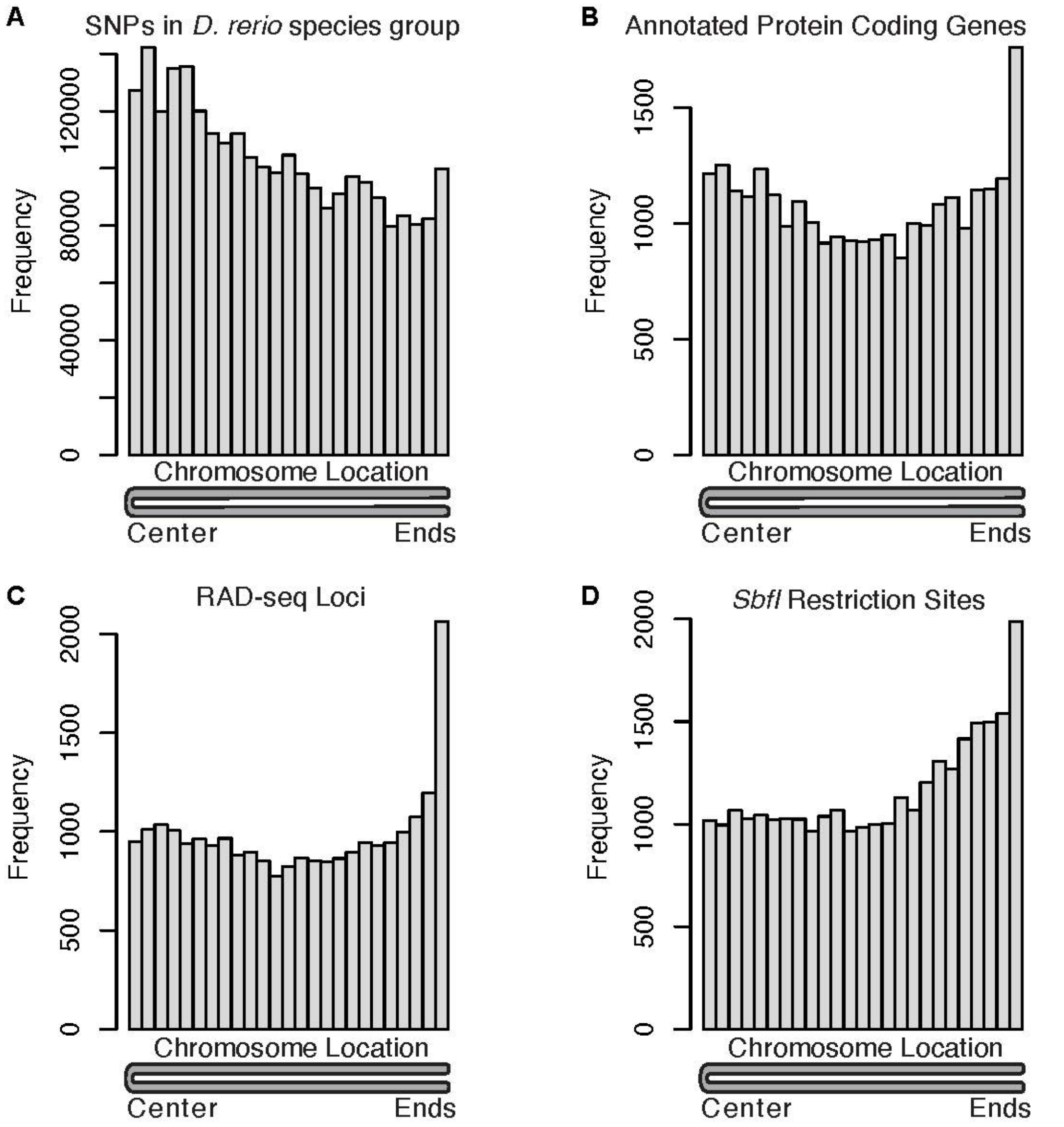
A
SNPs in D. rerio species group
Frequency
0
40000
80000
120000
Chromosome Location
Center
Ends
B
Annotated Protein Coding Genes
Frequency
0
500
1000
1500
Chromosome Location
Center
Ends
C
RAD-seq Loci
Frequency
0
500
1000
1500
2000
Chromosome Location
Center
Ends
D
SbfI Restriction Sites
Frequency
0
500
1000
1500
2000
Chromosome Location
Center
Ends

Supplemental Figure S3: Interspecific Shared Derived Characters in *D. rerio* populations

Violin plots show the distribution of shared derived characters (SDCs) according to chromosome location. SDCs are either shared by *D. kyathit* and *D. rerio* (in green) or *D. aesculapii* and *D. rerio* (in orange). The distribution of sites identified in this study (Exome SDCs) are shown without points. The sites found in both this study and previous RAD-seq studies are shown with points and split according to whether the SDCs were variable or fixed across all fish.

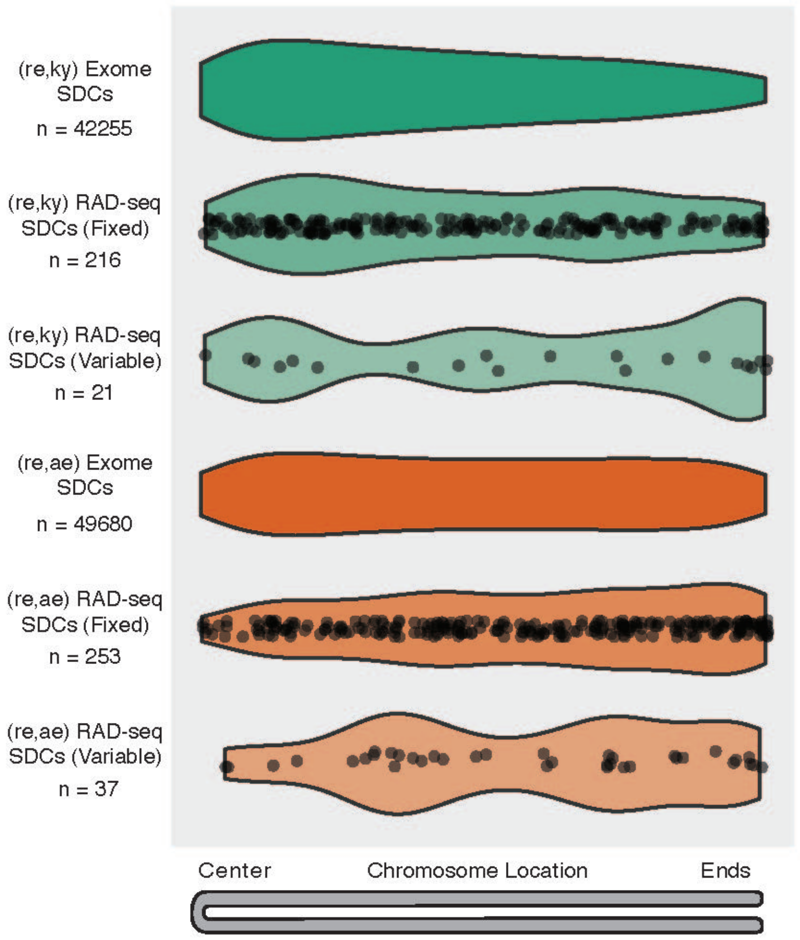
(re,ky) Exome SDCs
n = 42255
(re,ky) RAD-seq SDCs (Fixed)
n = 216
(re,ky) RAD-seq SDCs (Variable)
n = 21
(re,ae) Exome SDCs
n = 49680
(re,ae) RAD-seq SDCs (Fixed)
n = 253
(re,ae) RAD-seq SDCs (Variable)
n = 37
Center
Chromosome Location
Ends

Supplemental Table S1. Illumina library statistics

Species	Total Reads	Reads Aligned to GRCz10	Proportion Aligned	Exome Coverage
Danio rerio	41603616	41292106	0.993	87.839
Danio aesculapii	35171505	31812385	0.904	67.673
Danio kyathit	50412319	45793130	0.908	97.414
Danio nigrofasciatus	37389413	33110288	0.886	70.434
Danio albolineatus	31916307	27771158	0.87	59.077
Danio kerri	39820645	34829199	0.875	74.091
Danio choprae	52038977	45517922	0.875	96.829
Danio erythromicron	25080033	20885438	0.833	44.429
Danio margaritatus	35758836	30482592	0.852	64.845
Danio meghalayensis	39328467	33221487	0.845	70.671
Devario aequipinnatus	31491855	22986682	0.73	48.899

Supplemental Table S2: Validation of species identification and method robustness from BLAST results for rho and rag1 nucleotide sequences for Danio and Devario available in public databases, when compared to sequences reconstructed in this study.

Reconstructed Exome Sequence	Accession number	Percent Identity	Alignment Length	Accession Species	Description
Drer_rho_ENSDART00000027000	LR812070.1	100	467	Danio rerio	Danio rerio genome assembly, chromosome: 8
Daes_rho_ENSDART00000027000	EU241365.1	99.399	333	Danio aesculapii	Danio sp. 'snakeskin' rhodopsin (Rho) gene, partial cds
Dkya_rho_ENSDART00000027000	LR812534.1	99.786	467	Danio kyathit	Danio kyathit genome assembly, chromosome: 16
Dnig_rho_ENSDART00000027000	HM223909.1	100	317	Danio nigrofasciatus	Danio nigrofasciatus isolate CTOL01597 rhodopsin gene, partial cds
Dalb_rho_ENSDART00000027000	HQ286331.1	98.738	317	Danio albolineatus	Danio albolineatus voucher 7205 rhodopsin photopigment (rh1) mRNA, partial cds
Dker_rho_ENSDART00000027000	JQ614125.1	100	320	Danio kerri	Danio kerri rhodopsin (Rho) gene, partial cds
Dcho_rho_ENSDART00000027000	JQ614130.1	99.688	320	Danio choprai	Danio choprai voucher RC0060 rhodopsin (Rho) gene, partial cds
Dery_rho_ENSDART00000027000	HM223905.1	100	285	Danio erythromicron	Microrasbora erythromicron isolate CTOL00425 rhodopsin gene, partial cds
Dmar_rho_ENSDART00000027000	JQ614141.1	99.688	320	Danio margaritatus	Danio margaritatus voucher RC0107 rhodopsin (Rho) gene, partial cds
Dmeg_rho_ENSDART00000027000	JQ614143.1	100	320	Danio meghalayensis	Danio meghalayensis voucher RC0565 rhodopsin (Rho) gene, partial cds
DEVaeq_rho_ENSDART00000027000	JQ614155.1	98.125	320	Devario aequipinnatus	Devario aequipinnatus voucher RC0352 rhodopsin (Rho) gene, partial cds
ABZ_rag1_ENSDART00000142917	LR812087.1	96.122	1083	Danio rerio	Danio rerio genome assembly, chromosome: 25
Daes_rag1_ENSDART00000142917	LR812502.1	96.399	1083	Danio aesculapii	Danio aesculapii genome assembly, chromosome: 25
Dkya_rag1_ENSDART00000142917	LR812543.1	96.214	1083	Danio kyathit	Danio kyathit genome assembly, chromosome: 25
Dnig_rag1_ENSDART00000142917	EU292699.1	96.781	994	Danio nigrofasciatus	Danio nigrofasciatus isolate CTOL01597 recombination activating protein 1 (RAG1) gene, exon 3 and partial cds
Dalb_rag1_ENSDART00000142917	EU292696.1	96.329	1008	Danio albolineatus	Danio albolineatus isolate CTOL01500 recombination activating protein 1 (RAG1) gene, exon 3 and partial cds
Dker_rag1_ENSDART00000142917	HM224029.1	96.925	1008	Danio kerri	Danio kerri isolate CTOL02792 recombination activating protein 1 (RAG1) gene, partial cds
Dcho_rag1_ENSDART00000142917	HM224026.1	97.805	410	Danio choprai	Danio choprai isolate CTOL01667 recombination activating protein 1 (RAG1) gene, partial cds
Dery_rag1_ENSDART00000142917	EU292698.1	98.28	407	Danio erythromicron	Microrasbora erythromicron isolate CTOL01953 recombination activating protein 1 (RAG1) gene, exon 3 and partial cds
Dmar_rag1_ENSDART00000142917	EU292695.1	98.034	407	Danio margaritatus	Danio margaritatus isolate CTOL02780 recombination activating protein 1 (RAG1) gene, exon 3 and partial cds
Dmeg_rag1_ENSDART00000142917	EU292697.1	98.025	405	Danio dangila	Danio dangila isolate CTOL01501 recombination activating protein 1 (RAG1) gene, exon 3 and partial cds
DEVaeq_rag1_ENSDART0000014291[illegible]	KJ548110.1	94.729	645	Devario aequipinnatus	Devario aequipinnatus isolate 5 recombination activating protein 1 (RAG1) gene, partial cds

Supplemental Table S3: HyDe Analyses

Location	Zscore	Pvalue	Gamma
Whole Genome	12.0228601	0	0.20614183
Center 20%	8.15554143	2.22E-16	0.52007463
Semi Center 20%	8.60309921	0	0.478127
Medial 20%	6.80299617	5.15E-12	0.33951127
Semi End 20%	0.95477042	0.1698469	0.05357375
End 20%	0.20771587	0.41772544	0.00427926
